# ﻿*Mazama tschudii* (Wagner, 1855), forgotten by science, re-emerges as a new genetic lineage of Neotropical deer with a proposed neotype (Artiodactyla, Cervidae)

**DOI:** 10.3897/zookeys.1265.157429

**Published:** 2025-12-23

**Authors:** Eluzai Dinai Pinto Sandoval, José Eduard Hernández-Guevara, Agda Maria Bernegossi, Pedro Henrique Faria Peres, Renato Caparroz, José Maurício Barbanti Duarte

**Affiliations:** 1 Núcleo de Pesquisa e Conservação de Cervídeos (NUPECCE), Departamento de Zootecnia, Faculdade de Ciências Agrárias e Veterinárias, Universidade Estadual Paulista (UNESP), Jaboticabal-SP, Brazil Universidade Estadual Paulista (UNESP) Jaboticabal Brazil; 2 Departamento Académico de Ciencias Pecuarias, Universidad Nacional Agraria de la Selva, Carretera Central Km 1.21, Tingo María, Peru Universidad Nacional Agraria de la Selva Tingo María Peru; 3 Departamento de Genética e Morfologia, Instituto de Ciências Biológicas, Universidade de Brasília (UnB), Brasília 70910-900, Distrito Federal, Brazil Universidade de Brasília (UnB) Distrito Federal Brazil

**Keywords:** Brocket deer, cytogenetics, mitochondrial DNA, phylogeny, taxonomy

## Abstract

The accurate classification of Neotropical deer is essential for effective conservation strategies; however, many species within the genus *Mazama* remain taxonomically unresolved due to morphological similarities and historical uncertainties. *Mazama tschudii*, originally described from the western Andes of Peru, has been debated due to the absence of a designated type specimen and its resemblance to other brocket species. This study integrates morphological, cytogenetic, and molecular data to clarify its taxonomic status, designate a neotype, and highlight its significance for conservation. A recently collected specimen from La Ramada, Lambayeque, Peru, matched the original description of Wagner (1855), exhibiting a smaller size, darker pelage, and distinct cranial features compared to closely related species such as *Mazama americana*, *M. temama*, and gray brockets (*Subulo gouazoubira*, *Passalites nemorivagus*). Cytogenetic analysis revealed a diploid number of 2n = 42 and a fundamental number (FN) of 68, with extensive chromosomal rearrangements that distinguish it from other *Mazama* species and suggest reproductive isolation. Mitogenome analysis placed *M. tschudii* as a sister species to *M. temama*, yet distinct from *M. americana*. Despite this close molecular relationship with *M. temama*, chromosomal divergence and BAC-FISH results demonstrated independent evolutionary trajectories, with ten centric and four tandem fusions differentiating their karyotypes. These results provide robust chromosomal and genomic evidence to validate *M. tschudii* as a distinct species under the biological species concept. The formal neotype designation from its historical type locality establishes a definitive taxonomic reference, contributing critical insights into the evolutionary complexity of Neotropical deer and reinforcing the importance of integrative taxonomy in shaping conservation priorities.

## ﻿Introduction

The genus *Mazama* Rafinesque, 1817 is composed by small to medium-sized deer with simple, unbranched antlers, distributed from Mexico to Argentina. Its taxonomy was initially based on external morphological characteristics, leading to 42 nominal taxa; however, currently, only six valid species are recognized: *M. americana*, *M. rufa*, *M. rufina*, *M. temama*, *M. jucunda*, and *M. nana* (González and Duarte 2020; Heckeberg 2020; Peres et al. 2021). Among these, *M. rufina* has been discussed as representing a distinct lineage, and even proposed as deserving recognition under a separate genus (Gutiérrez et al. 2017).

Historically, species within *Mazama* were classified into two groups, red and gray brockets, based on cranial and body skin characteristics (Allen 1915). However, different authors have cited that morphology is not an efficient taxonomic criterion for the genus due to the great morphological similarity among brocket deer species, related to evolutionary convergence (Groves and Grubb 1987; Merino and Rossi 2010) and recent divergence (Duarte et al. 2008; Peres et al. 2021). Thus, cytogenetic and genetic approaches have been more efficient in resolving taxonomic uncertainties within this genus (Duarte et al. 2008; Heckeberg et al. 2016; Bernegossi et al. 2023). The study of chromosomes is particularly relevant, as numerical or structural differences may cause reproductive barriers, resulting in infertile offspring (Cursino et al. 2014; Salviano et al. 2017; Galindo et al. 2021). This could be used to distinguish species based on the biological species concept, reinforcing the importance of cytogenetic approaches in the taxonomy of this group (Coyne and Orr 1998; Peres et al. 2021). Additionally, phylogenetic and evolutionary species concepts (Cracraft 1989; Wiley and Mayden 2000), commonly applied in molecular systematics, also provide valuable frameworks by identifying lineages based on shared ancestry and diagnosable differences. These perspectives are particularly useful in recent or cryptic radiations, such as in *Mazama*. Therefore, integrative taxonomy, which combines morphological, cytogenetic, and molecular data, offers a comprehensive approach for robust species delimitation in this complex genus.

Recent taxonomic rearrangements have been made for some Neotropical deer like *Mazama americana* (Erxleben, 1777), *Mazama temama* (Kerr, 1792), *Subulo gouazoubira* (Fischer, 1814), formerly *Mazama gouazoubira*, *Passalites nemorivagus* (Cuvier, 1817), formerly *Mazama nemorivaga*, *Odocoileus pandora* (Merriam, 1901), formerly *Mazama pandora*, based on genetic studies (Escobedo-Morales et al. 2016; Cifuentes-Rincón et al. 2020; Peres et al. 2021; Sandoval et al. 2022; Bernegossi et al. 2023; Escobedo-Morales et al. 2025). However, a review of the genetic identity of other species, which were previously described but later synonymized with the aforementioned ones based solely on morphological traits, is still lacking. This is the case of the Peruvian brocket deer described in 1855 by Johan A. Wagner, based on a specimen collected by the Swiss naturalist Johann Jakob von Tschudi (1818–1889) from the western Andes in the coastal region of Peru. Initially, Wagner had referred to this deer as *Cervus nemorivagus* in 1844, due to its grayish coloration, aligning it with the species known from French Guiana and Brazil. However, upon further examination of the specimen’s distinct morphological traits and its geographically distant Andean origin, Wagner recognized it as a separate taxon and proposed the new name combination *Cervus tschudii*. Among the morphological criteria considered to distinguish this species from *P. nemorivagus*, Wagner (1855) cited the brown coloration on the dorsum with whitish speckles of individual hairs that are brown along the length but become lighter at the tips, slightly darker coloration in the head around the eyes, and slightly larger in size than the Brazilian gray brocket species. According to the author, this deer is found in the hilly regions of the Andes Mountain range, occurring throughout the Western Cordillera up to approximately 16,000 feet in Peru (Wagner 1855).

In the decades after its description, taxonomic reviews concerning *Cervus tschudii* were inconsistent. While the species is listed in the work of Fitzinger (1873), it is ignored in the review by Brooke (1878), who only considered the red brocket *Coassus whitelyi* Gray, 1873, to be present in Peru. Lydekker (1898) examined both descriptions of *C. tschudii* Wagner, 1855 and *C. whitelyi* Gray, 1873, classifying the latter as a junior synonym of *Mazama tschudii*. Additionally, the author observed that the dorsal pelage was darker, and the lower parts and the inner sides of the limbs were pure white rather than yellowish-white when compared to the Amazon brown brocket *M. nemorivaga* [= *Passalites nemorivagus* (Cuvier, 1817)] (Lydekker 1898). Allen (1915) referred to *M. tschudii* as part of the brown brocket group and argued that it differs significantly in coloration and size from *Cervus simplicicornis*. In this regard, the author proposed the possible occurrence of various forms of this type in Peru, suggesting that a new taxonomic review would be needed to determine the type locality (Allen 1915). Following Wagner (1855), Lydekker (1898), and Allen (1915), no published studies on morphology, nomenclature, or genetics have confirmed the taxonomic identity of the Peruvian brockets in the western Andes. Besides the synonymy of *Mazama tschudii* and *M. whitelyi*, specimens from this region have been considered as a variant of the Amazon gray brocket deer *P. nemorivagus* (Cuvier, 1817) (Rossi et al. 2010). Therefore, it is necessary to assign a type with a known origin in the wild to perform an integrative study based on morphological and genetic characteristics of the species. Our field sampling was thus specifically designed to locate and evaluate a gray brocket individual from the western Andes Mountain in Peru, consistent with Wagner’s original description, both in terms of geography and general morphology. Given the absence of an extant holotype or reliably identifiable original material, designating a neotype from the recently collected specimen is crucial to stabilizing the species’ taxonomy and providing a reference for future research.

Thus, the objective of this study was to characterize the morphology (body biometrics, coloration patterns, cranial and post-cranial characterization), cytogenetics (conventional karyotype, chromosomal biometrics, C-banding, Ag-NOR staining, and FISH), and phylogenetic position (mitochondrial DNA) for *Mazama tschudii*, while evaluating the species’ validity and supporting the formal designation of a neotype to ensure taxonomic clarity and facilitate future comparative studies.

## ﻿Materials and methods

### ﻿Study area and sample collection

**Figure 1.**
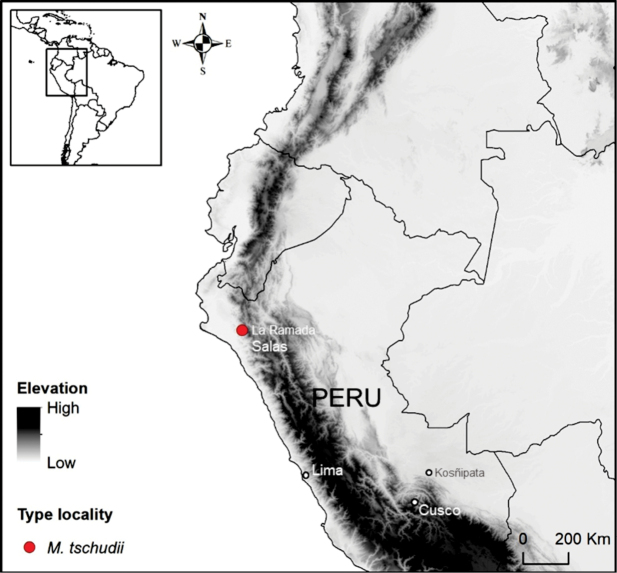
Collection locality of the neotype for *Mazama tschudii* (T431/MHNC008-My), from Lambayeque, northwestern Andes Mountain, Peru (red circle). The gray-shaded area indicates the extent of the Andean Cordillera.

The permit for sample collection was obtained from the Servicio Nacional Forestal y de Fauna Silvestre (**SERFOR**) in Peru, and a scientific expedition was organized in the Western Andes Mountain range of the country. A male individual was collected from the locality of La Ramada, Salas District, Lambayeque Province, Peru, on 22 January 2020 (Fig. 1). The collection site coordinates were 6°24'57"S, 79°43'1"W, at an altitude of 1,510 meters above sea level. The specimen was assigned the identification number T431 by the Deer Research and Conservation Center (NUPECCE) and deposited at the Natural History Museum of the National University of San Antonio Abad del Cusco under number MHNC008-My.

### ﻿Morphological characterization

After collection, external body measurements were taken to gather data on weight, length of the left and right antlers, diameter at the base of the right and left antlers, head length, head width, ear size, distance between eyes, distance between antlers, mandibular width at the base, metacarpal length, metatarsal length, height, body length, tail length, neck girth, thoracic girth, and abdominal girth. These measurements were taken using a scale, measuring tape, and calipers.

The individual was also positioned on a blue field cloth with metric scales, and photographs were taken in lateral, dorsal, and ventral views, as well as close-ups of the head. The skin was removed entirely from the underlying muscular tissue, fat, and fascia. It was then immersed in a tanning solution to desiccate the material. After drying for three days, the skin was kept for posterior morphological studies.

The pelage coloration and chromogenetic fields were examined on both the skin and photographs. The following features were assessed following Rossi (2000) and Hershkovitz (1982): general pelage coloration; chromogenetic fields of the head, including upper and lower orbital bands, anterior superciliary patch, anterior and posterior auricular regions, rostral band, nasal region, mental region, mandibular patch, buccal region, and gular region; and chromogenetic fields of the body, head and neck region, dorsal midline of the body, dorsal tail, posterior dorsal region of the body, ventral region of the body, underside of the tail, distal limb region, and pigment band patterns in the hairs of different body regions. Additionally, the presence of reverted hair bands in the neck and a rounded tuft of hair in the tarsal region was noted. The complete skeleton, including the skull and post-cranial elements, was cleaned at the collection site and stored in plastic boxes at room temperature. Thirty-four standard body and skull measurements, following von den Driesch (1976), were recorded (Suppl. materials 1, 2).

The collected specimen was examined to confirm the presence or absence of tarsal and metatarsal hair tufts, to describe the pelage coloration, and the profile of the nasal bridge (e.g., arched vs. straight), features mentioned in the original description of *Mazama tschudii* (Wagner 1855; Lydekker 1898). Then, the morphological data from the specimen described here were compared to recent descriptions of other Neotropical deer species, including *Subulo gouazoubira* (González and Duarte 2020; Bernegossi et al. 2023), *Passalites nemorivagus* (Morales-Donoso et al. 2023), *Mazama americana* (Cifuentes-Rincón et al. 2020), *Mazama rufa* (Peres et al. 2021), and *Mazama temama* (Sandoval et al. 2022; 2024). These comparisons served to contextualize the observed traits within the known variation across Neotropical deer taxa and contributed to the identification and validation of species-level diagnostic characters for the collected specimen.

### ﻿Cytogenetic characterization

A 5 × 2 cm fragment of skin from the inguinal region of the topotype was collected and divided into ten parts, which were then placed in a specific culture medium as described by Duarte et al. (2021). The samples were then refrigerated at 4 °C for 3 h, exposed to liquid nitrogen vapor for 30 min, and subsequently submerged in liquid nitrogen at -196 °C for storage. The skin samples were thawed in a water bath at 37 °C, transferred to flasks containing culture medium enriched with fetal bovine serum, and incubated at 37 °C with 5% CO_2_. After achieving cell confluence, the material underwent colchicine treatment, followed by a hypotonic solution, and was fixed in Carnoy’s solution (Verma and Babu 1995). Chromosome classification was conducted using ten metaphases from the collected topotype. The chromosomes were classified based on arm length ratios as metacentric, submetacentric, or acrocentric (Levan et al. 1964). Relative length (RL) was used to group chromosome pairs into the following categories: group A (large two-armed chromosomes, RL≥6%), group C (small two-armed chromosomes, RL<6%), group D (large one-armed chromosomes, RL≥5%), group E (small one-armed chromosomes, RL<5%), and group B (B chromosomes, RL<1.5%) (Cifuentes-Rincón et al. 2020). Additionally, C-banding (Sumner 1972) was performed to visualize constitutive heterochromatin regions, and Ag-NOR staining (Howell and Black 1980) was used to identify nucleolar organizer regions.

Fluorescence in situ hybridization (FISH) was performed on metaphases of the skin cultures using Bacterial artificial chromosome (BAC) clones from the bovine CHORI-240 library (BACPAC Genomics, Emeryville, CA, USA), based on NCBI ARS-UCD1.2 genome assembly data. The probes used were selected according to Bernegossi et al. (2022). BAC probe mapping was used to analyze chromosomal rearrangements in the species, with *Subulo gouazoubira* (SGO) (2n = 70 and FN = 70) serving as a reference due to its retention of the putative basal karyotype. This species has also been consistently used for cytogenetic comparisons within the group (Bernegossi et al. 2022).

For the DNA extraction, an adapted protocol from the Wizard Plus SV Minipreps DNA Purification Systems method was used. BAC DNA was labeled with biotin-16-dUTP or digoxigenin-11-dUTP (Roche, Mannheim, Germany), using the BioPrime Array CGH Genomic Labeling kit (Invitrogen, Carlsbad, CA, USA). The FISH procedure followed the protocol described by Vozdova et al. (2019). A Zeiss AxioCam RM camera attached to an Olympus BX60 microscope (100× objective), equipped with appropriate fluorescence filters, was used to visualize the FISH results.

The karyotype of the collected specimen was compared with cytogenetic data previously published for other Neotropical deer species Abril and Duarte 2008; Peres et al. 2021; Duarte and Jorge 2003; Morales-Donoso et al. 2023; Sandoval et al. 2024). The comparison includes diploid number (2n), fundamental number (FN), classical banding patterns, and FISH (fluorescence in situ hybridization) results.

### ﻿DNA extraction and mitochondrial DNA sequencing

After collecting the specimen, liver and muscle tissues were sampled and used for DNA analysis. Genomic DNA was extracted from tissue samples using the commercial DNeasy Blood and Tissue kit (Qiagen, Valencia, CA, USA), which involved digestion with proteinase K and silica column extraction and purification, following the manufacturer’s protocol. The DNA extractions were quantified using a Qubit fluorometer and subsequently diluted to a working solution (50 ng/µl) for sequencing. The DNA sample from the collected specimen from Peru was sheared by Covaris sonication to an average size of 500 base pairs (bp). Then, this fragmented DNA was used as input for genomic library preparation and indexing using the TruSeq® Nano DNA Library prep kit and Illumina Unique Dual Indexes, following the manufacturer’s instructions. The genomic library was quantified through qPCR for pooling in equimolar ratio on a shared run and subjected to a shotgun next-generation sequencing (2×100 bp) in an Illumina HiSeq 2000. The sequencing resulted in 17M reads that were mapped to to the reference mitogenome of *P. nemorivagus* (JN632659) following the “medium-low sensitivity” parameters in Geneious Prime 2023.1.1. The mitogenome was successfully recovered with at least 10-fold coverage per base. For the phylogenetic analysis of the mitogenome of the Peruvian specimen retrieved in this study, sequences from other Neotropical deer species were downloaded from the online GenBank database (Table 1). *Alces alces* was used as an outgroup in the phylogenetic analyses.

**Table 1.** Mitochondrial DNA sequences from Neotropical cervid species used in the phylogenetic analysis.

Species	GenBank accession number	Origin (Country – Locality)
*Alces alces*	MF784602	
*Blastocerus dichotomus*	JN632603	Bolivia
*Mazama americana*	JN632657	Peru
*Mazama americana*	JN632656	French Guiana
*Mazama americana* – Neotype	MZ350857	French Guiana
*Mazama jucunda*	MZ350859	Brazil
*Mazama nana*	MZ350863	Brazil
*Mazama rufa* – Neotype	OQ198444	Brazil
*Mazama rufina*	JN63266	Colombia
*Mazama temama*	JN632673	Colombia
*Mazama temama* – Neotype	MZ350864	Mexico
*Mazama temama*	MZ362858	Mexico
*Mazama tschudii* – Neotype	PV299131	Peru, La Ramada
*Odocoileus pandora*	OQ731409	Mexico
*Odocoileus pandora*	OQ731408	Mexico
*Odocoileus virginianus*	KM612273	USA, Texas
*Odocoileus virginianus*	KM612271	Mexico, Veracruz
*Ozotoceros bezoarticus*	JN632681	Bolivia
*Ozotoceros bezoarticus*	MZ350860	Brazil
*Passalites nemorivagus*	JN632659	Peru
*Passalites nemorivagus*	MZ350867	Brazil
*Subulo gouazoubira*	MZ350866	Brazil
*Subulo gouazoubira* – Neotype	MZ350858	Paraguay

### ﻿Phylogenetic analysis

The sequences were aligned using the MAFFT 7 tool (Katoh et al. 2019). We partitioned the mitogenomes matrix into 63 blocks, considering 22 tRNAs, 2 rRNAs, and the three codon positions of the 13 protein-coding genes. The control region was excluded from the analysis due to its high mutation, deletion, and insertion ratios that compromised the alignment quality and promoted some level of saturation in previous studies (Peres et al. 2021). The best nucleotide substitution models were selected by PartitionFinder 2.1.1 (Lanfear et al. 2017) under the Akaike Information criterion, using available MrBayes models. Phylogenetic analysis was performed using Bayesian Inference (BI) in the MrBayes on XSEDE 3.2.1 program (Huelsenbeck and Ronquist 2001), available as a web service on the CIPRES Science Gateway platform (Miller et al. 2010), with 25,000,000 generations until reaching a variance <0.01. Metropolis-Coupled MCMC (Markov Chain Monte Carlo) was used to estimate the posterior probability distribution, with four chains, two independent runs, and a 25% burn-in of the initial samples. The majority rule consensus tree obtained from the analysis was edited using the FigTree 1.4.0 software (Rambaut 2012).

## ﻿Results

### ﻿Morphological characterization

**Figure 2.**
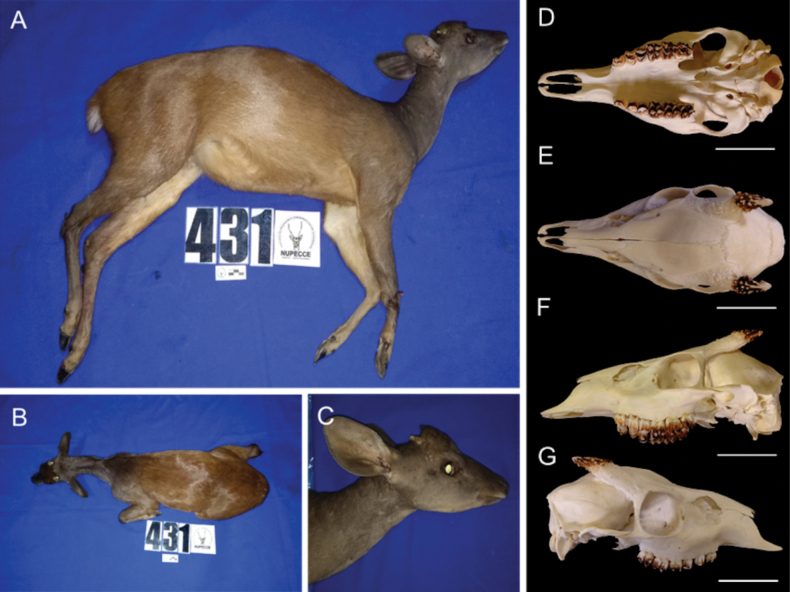
Male specimen of *Mazama tschudii* (T431/MHNC008-My), collected in Lambayeque, Northwestern Cordillera of Peru. **A.** Lateral view of the body; **B.** Dorsal view of the body; **C.** Lateral view of the head; **D.** Skull ventral view; **E.** Skull dorsal view; **F.** Skull lateral left view; **G.** Skull lateral right view. Scale bars: 2 cm.

The collected specimen in this study (Catalog number T431/MHNC008-my, Natural History Museum, National University of San Antonio Abad del Cusco) exhibited an overall brownish coloration with a dark brown dorsal-medial stripe extending to the dorsal region of the tail. The ventral region of the body was pale yellowish and whitish in the inguinal region, with long white hairs on the ventral side of the tail. Long hairs were observed on the ventral chest region, with no hair in the inguinal area. The dark gray coloration of the neck contrasted with the rest of the body. The hind limbs were brown laterally, with reddish hairs in the lumbar area and gray hairs in the metatarsal region. There was an absence of tarsal and metatarsal tufts. The forelimbs were brown laterally and dark gray distally. Similarly, the forelimbs appeared brown laterally and gray in the metacarpal region, with the ventral region ranging from yellowish to whitish. The external region of the ears was dark gray, similar to the neck, and the internal region was dark brown, with a white base and no hair at the base of the ear. The orbital region was dark gray, overlaid with a yellowish band, and featured a yellowish lacrimal opening. The chin region was dark brown, gradually lightening ventrally toward the neck along the ventral midline. Additionally, the specimen exhibited a white nasal patch and a frontal tuft (Fig. 2).

The skull presented short, simple, and longitudinally grooved antlers with a length of 1.8 cm (right) and 2.1 cm (left), a medium-sized auditory bulla, and two orbital foramina along with an oval-shaped preorbital fossa (Fig. 2).

The specimen of *M. tschudii* exhibited a head length of 7.5 cm, a head width of 9.6 cm, ear length of 9.6 cm, an eye spacing of 4.4 cm, mandible width of 6.3 cm, height of 51 cm, body mass of 13.6 kg, and metacarpus and metatarsus lengths of 11 cm and 19.1 cm, respectively. In terms of cranial measurements, the specimen had a total length of 174.7 mm, an akrokranium measurement of 120.6 mm, a cheektooth row length of 55.87 mm, a zygomatic breadth of 72.6 mm, and a greatest breadth across the orbits of 73.15 mm. See complementary measurements in Suppl. materials 1, 2.

### ﻿Cytogenetic characterization

**Figure 3.**
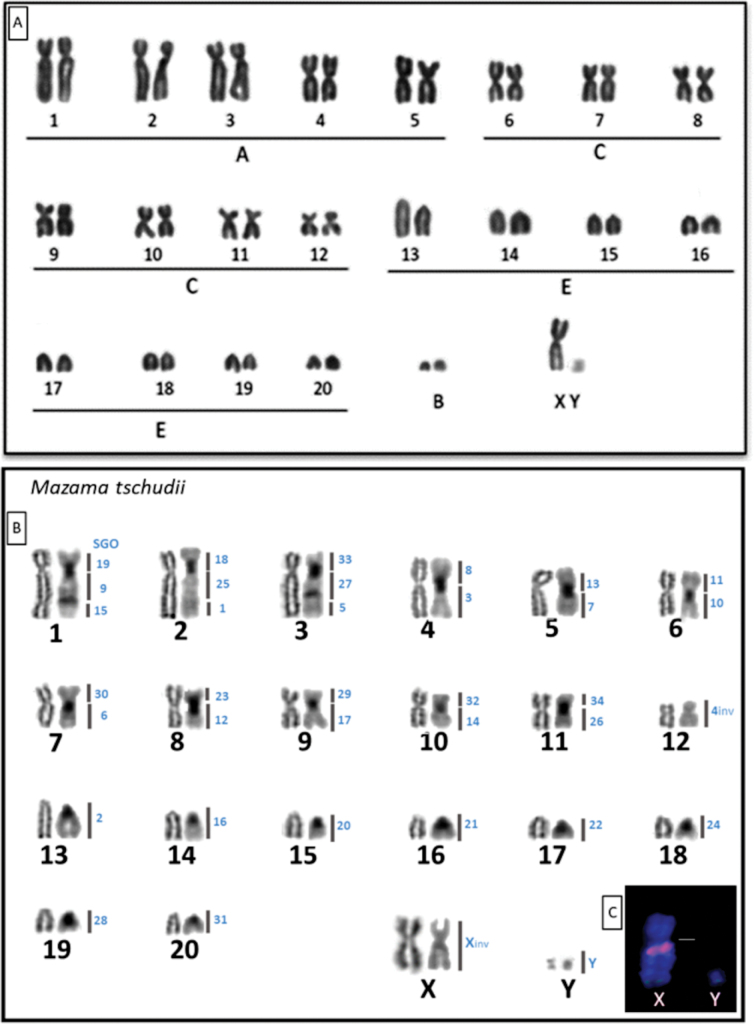
Karyotype of a male specimen of *Mazama tschudii* (T431/MHNC008-My), collected in Lambayeque, Northwestern Andean Cordillera of Peru. Diploid number (2n) = 42, fundamental number (FN) = 68, XY, B chromosome 0–3. **A.** Giemsa Conventional staining; **B.** From left to right, Ag-RON staining; C Banding, homologies with *Subulo gouazoubira* chromosomes; **C.**FISH of sex chromosomes, BAC311B9 (red) and BAC453C5 (green). Abbreviations: cs = centromere shift, inv = inversion.

The collected specimen of *M. tschudii* presented a diploid number (2n) of 42 and a fundamental number (FN) of 68. Twelve pairs of bi-armed autosomal chromosomes and eight pairs of acrocentric autosomal chromosomes were observed. Chromosome classification by relative length placed pairs 1–5 in Group A (large bi-armed chromosomes), pairs 6–12 in Group C (small bi-armed chromosomes), and pairs 13–20 in Group E (small one-armed chromosomes). The X chromosome exhibited submetacentric morphology, while the Y chromosome was identified as a small submetacentric chromosome, the smallest in the set. One to two B chromosomes were also observed (Fig. 3).

In the metaphases analyzed under Ag-NOR staining, nucleolar region markings were observed on the telomeres of the long arm (q) of both chromosomes in pair 2, characterized as the second-largest bi-armed chromosome, and on the telomeres of the long arm (q) of the chromosomes in pair 13, characterized as the largest acrocentric chromosome (Fig. 3). C-banding revealed regions of constitutive heterochromatin associated with the centromere of all autosomal chromosomes, except for the smallest bi-armed chromosome, positioned as pair 12 in Fig. 2. Additionally, interstitial heterochromatin markings were observed on the long arms (q) of the large submetacentric chromosomes, corresponding to pairs 1–3 in Fig. 3.

The BAC probe mapping indicated that the karyotype divergence in *M. tschudii* primarily results from tandem fusions and centric fusions. Additionally, a centromere shift in SGO pair 4 resulted in the submetacentric morphology of *M. tschudii* pair 12. The X chromosome of the *M. tschudii* individual exhibited submetacentric morphology associated with an inversion in the proximal region of the acrocentric SGO X. The distal region maintained the same hybridization pattern observed in the distal region of the X chromosome in *S. gouazoubira* (Table 2).

**Table 2.** Chromosomal correspondence between *Mazama tschudii* (MTS) and *S. gouazoubira* (SGO).

MTS chromosomal pairs	SGO orthologues	MTS chromosomal pairs	SGO orthologues
1p	19	9p	29
1q	9,15	9q	17
2p	18	10p	32
2q	25,1	10q	14
3p	33	11p	34
3q	27,5	11q	26
4p	8	12	4
4q	3	13	2
5p	13	14	16
5q	7	15	20
6p	11	16	21
6q	10	17	22
7p	30	18	24
7q	6	19	28
8p	23	20	31
8q	12	X	X

Compared to other Neotropical deer species, *Mazama tschudii* exhibits a unique karyotypic pattern that distinguishes it from the gray brocket species *Subulo gouazoubira* and *Passalites nemorivagus*, as well as from the red brockets *Mazama americana* and *Mazama rufa*. Additional differences are also observed in comparison with *Mazama jucunda* and *Mazama nana*. A summary of the comparative cytogenetic data is provided in Table 3.

**Table 3.** Diploid number (2n) and fundamental number (FN) for *Mazama tschudii* and other Neotropical deer species.

Species	2n	FN	Reference
*Mazama americana*	45	51	Cifuentes-Rincón et al. 2020
*Mazama jucunda*	32–34	46	Duarte and Jorge 2003
*Mazama nana*	36–39	58	Duarte and Jorge 2003
*Mazama rufa*	52–53	56	Peres et al. 2021
*Mazama temama*	44	70	Sandoval et al. 2022
*Mazama tschudii*	42	68	This study
*Passalites nemorivagus*	66–70	70–72	Morales-Donoso et al. 2023
*Subulo gouazoubira*	70	70	Bernegossi et al. 2023

**Figure 4.**
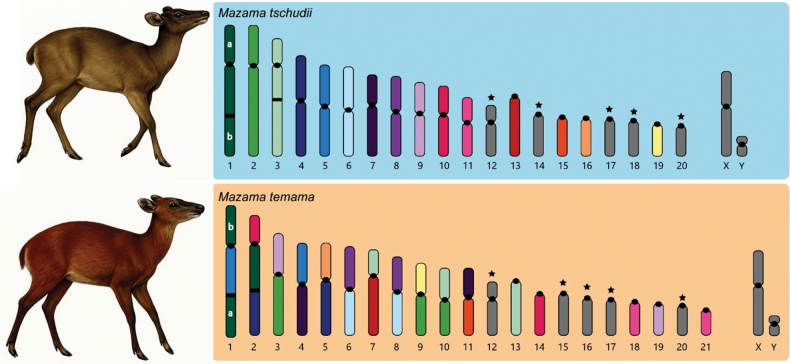
Comparative cytogenetics analysis of *Mazama tschudii* collected in Lambayeque, Northwestern Andean Cordillera of Peru (2n = 42 + 0 – 3 Bs, FN = 68) and the neotype of *M. temama* (2n = 44 + 0 – 4 Bs, FN = 70). The colored chromosomes indicate the karyotypes differences, each color representing one homologous chromosome. The stars indicate chromosomes that retain the structural configuration inferred from the hypothetical ancestral karyotype.

The karyotype of the collected specimen of *Mazama tschudii* (2n = 42, FN = 68) closely resembles that of the *M. temama* neotype (2n = 44, FN = 70) as described by Sandoval et al. (2022), based on classical staining. However, C-banding of *M. tschudii* revealed additional interstitial heterochromatic bands on the q-arms of one chromosomal pair that are not present in the *M. temama* neotype. Comparative analyses using BAC-FISH probes further distinguished the two species: the bi-armed chromosomes in *M. tschudii* differ in structure and composition from those in *M. temama* (Fig. 4). In particular, *M. tschudii* exhibits eleven centric and three tandem fusions not observed in *M. temama*, which itself has a distinct rearrangement pattern when compared to the ancestral karyotype of *Subulo gouazoubira* (2n = 70, FN = 70; Bernegossi et al. 2022). The only shared rearrangement between them is a centromere repositioning in ancestral chromosome pair 4, resulting in a submetacentric morphology of pair 12 and the presence of four non-fused acrocentric pairs. Fig. 4.

### ﻿Phylogenetic analysis

**Figure 5.**
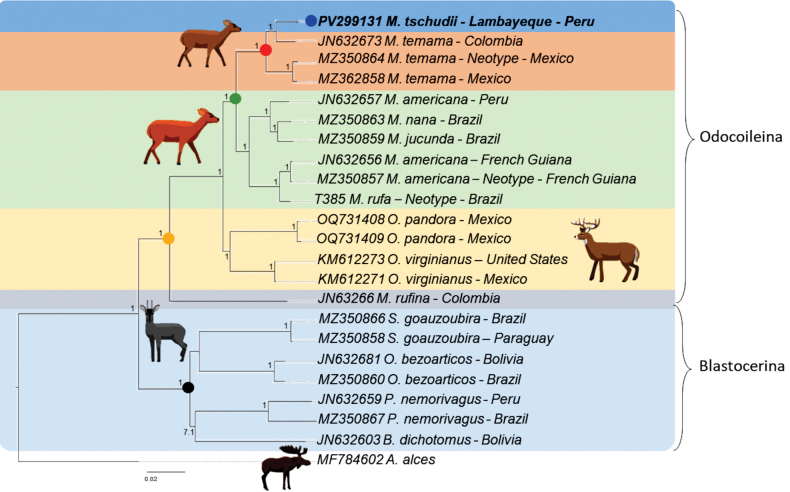
Bayesian inference tree of the mitogenomes from Neotropical species cervids. The value above the nodes represents the posterior probability of the analysis. Bold and blue circle: *Mazama tschudii* (T431/MHNC008-My) from Lambayeque, northwestern Cordillera of Peru; red circle: complex species cryptic *Mazama temama* clade; green circle: *Mazama species* clade; yellow circle: Odocoileina subtribe clade; black circle: Blastocerina subtribe clade.

The phylogenetic analysis of the complete mitogenomes positioned *M. tschudii* within the Odocoileina subtribe, alongside other *Mazama* species. Notably, the specimen was recognized as a sister taxon to *M. temama* from Colombia, belonging to the same clade as *M. temama* originating from Mexico. *M. americana* was recovered as paraphyletic, with individuals from French Guiana grouped in a sister clade to *M. rufa*, while a *Mazama americana* individual from Peru formed a sister clade with *M. jucunda* and *M. nana*. Our analysis also recovered genus *Odocoileus* as the sister group to *Mazama*, with high posterior probability (1). *Mazama rufina* was identified as the first to diverge within the Odocoileina clade. The genera *Subulo*, *Passalites*, *Ozotoceros*, and *Blastoceros* were also recovered within the subtribe Blastocerina, with high support (Fig. 5).

## ﻿Discussion

The description of *Cervus tschudii* Wagner, 1855, later included in the genus *Mazama* Rafinesque, 1817, was characterized by its darker coloration compared to other species of the genus. The morphological characters, such as the absence of tarsal and metatarsal tufts, and the arched nasal bridge cited by Wagner (1855) and Lydekker (1898) are consistent with those of the recently collected specimen. The cranial and body measurements also agree with the first descriptions of the species (Wagner 1855), revealing that *Mazama tschudii* is smaller than the red brockets, *Mazama americana* and *Mazama rufa*. It is important to note that Lydekker (1898) considered *M. tschudii* and the description of a red brocket specimen named *M. whitelyi* (Gray, 1873) as synonyms; however, this species was later synonymized with *M. americana* due to its larger skull (Cabrera 1960). Although no precise cranial measurements are available for *M. whitelyi*, the qualitative traits described by Gray, such as a larger and more ventricose braincase, rudimentary canines, and a narrower skull, are typical of red brockets in the *M. americana* complex (Rossi 2000). Therefore, this study confirms that size is the main difference between *M. tschudii* and the large red brocket species already described by other authors (Cifuentes-Rincón et al. 2020; Peres et al. 2021), and contrary to Lydekker’s (1898) assertion, *M. tschudii* is morphologically distinct from *M. americana*. In our study, the specimen of *M. tschudii* showed a larger body length (74.7 cm) and head length (24.5 cm) compared to the topotypes of *Passalites nemorivagus* and *Subulo gouazoubira*, which measured 70 cm and less than 20.5 cm of body and head length, respectively (Bernegossi et al. 2023; Morales-Donoso et al. 2023), suggesting there is also a size difference among these gray brocket species (Wagner 1855; Lydekker 1898). Compared to other small brocket species, such as *M. nana* and *M. temama*, the brownish skin coloration with darker areas on the neck and head is the primary difference between the recently collected specimen of *Mazama tschudii* and these small red brockets (Sandoval et al. 2022). Nevertheless, these comparisons are based on a limited sample size, and future studies incorporating broader quantitative and qualitative assessments of morphological differentiation are needed to substantiate these preliminary findings.

Our findings reinforce the value of cytogenetics and molecular approaches as valuable tools for advancing the taxonomic discussion of species within the genus *Mazama* (Abril et al. 2010; Cifuentes-Rincón et al. 2020; González and Duarte 2020). The karyotypic data obtained from our specimen contribute to a broader understanding of chromosomal variation within the genus, which is particularly relevant given the non-monophyletic condition of *Mazama* described by several authors (Hassanin et al. 2012; Gutiérrez et al. 2017; Heckeberg 2020).

Thus, the cytogenetic of the specimen of *Mazama tschudii* provides compelling evidence for its specific distinctiveness among Neotropical deer. The diploid number (2n = 42) and fundamental number (FN = 68) observed in *M. tschudii* are unique among described brocket deer, and notably distinct from the karyotypes of *Mazama americana*, *M. rufa*, *Subulo gouazoubira*, and *Passalites nemorivagus* (Abril and Duarte 2008; Cifuentes-Rincón et al. 2020; Peres et al. 2021; Bernegossi et al. 2023; Morales-Donoso et al. 2023). Regarding *M. temama*, although the phylogenetic analysis showed that *M. tschudii* clustered in the same clade with the Central American red brocket, comparative cytogenetic analyses revealed marked chromosomal divergence between the two taxa. Specifically, C-banding identified heterochromatic patterns not present in *M. temama*, and BAC-FISH results demonstrated that the bi-armed chromosomes in *M. tschudii* showed a different chromosomal composition from the karyotype of *M. temama* individuals from Mexico; with ten centric fusions and four tandem fusions distinguishing their karyotypes (Sandoval et al. 2024) from the hypothetically ancestral karyotype retained by *Subulo gouazoubira* (Bernegossi et al. 2022). Based on this, it is evident that they do not share the same chromosomal divergence history, despite their genetic closeness.

Evidence of chromosomal rearrangements supports species differentiation between *M. americana* and *M. rufa* (Peres et al. 2021), and has further indicated the existence of a complex cryptic species in *M. americana* (Bernegossi et al. 2024) and *M. temama* (Sandoval et al. 2024). Based on previous reproductive studies in the *M. americana* complex, along with new insights into chromosomal differences provided by molecular cytogenetics analysis, it is well-established that individuals with such discrepant differences in karyotypes, if crossed, would produce sterile offspring (Cursino et al. 2014; Salviano et al. 2017). Similarly, the karyotypic differences between *M. temama* (sensu stricto) and *M. tschudii* could act as effective reproductive barriers, compromising meiotic pairing between individuals of these species. In addition, it is important to consider the extensive intraspecific karyotypic variation observed within the *M. temama* complex (Sandoval et al. 2024). Across its distribution in Mexico, populations exhibit differences in diploid and fundamental numbers (2n = 42–50; FN = 68–70), with unique combinations of centric and tandem fusions distinguishing geographically separated populations such as those in Veracruz and Campeche. Even minor chromosomal differences, including a single tandem fusion or centric fusion in heterozygosis, can significantly compromise meiotic pairing, leading to increased rates of unbalanced gametes and reduced fertility (Salviano et al. 2017; Galindo et al. 2021). These rearrangements function as effective postzygotic reproductive barriers, despite limited morphological differentiation, illustrating how cryptic diversity can persist within morphologically similar populations. The cytogenetic divergence observed in *M. tschudii* relative to *M. temama* (sensu stricto) is of similar magnitude to the chromosomal difference between *M. rufa* and *M. americana*, reinforcing the species-level distinction of *M. tschudii* and supporting its recognition under the biological species concept. Taken together, these patterns underscore the crucial role of chromosomal differentiation in delimiting *Mazama* species boundaries and highlight the need for cytogenetic and integrative studies across other populations and lineages within *M. temama* complex to clarify the taxonomic status and inform conservation strategies (Sandoval et al. 2024; Escobedo-Morales et al. 2025).

The mitogenomes phylogenetic analysis placed *Mazama tschudii* within the Odocoileina subtribe, evidencing its phylogenetic dissociation from the gray brocket species *Subulo gouazoubira* and *Passalites nemorivagus*, which are positioned within the Blastocerina clade. This result contradicts the findings of different authors who have associated *M. tschudii* with these gray brockets (Allen 1915; Cabrera 1960; Rossi et al. 2010). Within Odocoileina, *M. tschudii* and *M. temama* formed a distinct and well-supported monophyletic clade, clearly separated from *M. americana* specimens sampled from diverse South American localities. Bayesian inference further supported this phylogenetic distinction, showing that *M. tschudii* does not cluster with any *M. americana* clades, including those featuring specimens from the type locality in French Guiana, and the clade that includes a Peruvian *M. americana* individual. Specifically, *M. tschudii* was found as the sister taxon to a specimen of *M. temama* from Colombia, diverging from other specimens of *M. temama* of Mexican origin. This Colombian *M. temama* sequence was initially discussed by Escobedo-Morales et al. (2016) using mitochondrial Cyt-b phylogeny, who identified phylogeographic structuring and notable genetic divergence between Mexican and Colombian populations. Subsequently, Gutiérrez et al. (2017) confirmed significant divergence of the Colombian *M. temama* from Mexican populations, suggesting it could represent an undescribed species. Both studies recommended revising the taxon through integrative studies of specimens from Colombia and Venezuela (Escobedo-Morales et al. 2016; Gutiérrez et al. 2017).

Besides the close relationship between the specimen *M. tschudii* collected from Peru and the Colombian *Mazama* sequence (JN632673), whether these individuals belong to the same species remains uncertain and requires additional sampling from both regions. A recent study (Escobedo-Morales et al. 2025) has demonstrated that *M. temama* represents a species complex composed of at least three independent lineages (*M. temama*, *M. reperticia*, and *M. zetta*), each of which requires separate taxonomic and conservation assessment. The authors further emphasized the importance of expanding geographic sampling and conducting integrative studies of these lineages to clarify their taxonomic status. While Escobedo-Morales et al. (2025) highlighted the role of next-generation sequencing and museum collections as key tools to refine our understanding of Neotropical deer evolutionary history, we stress that cytogenetic studies remain indispensable for reliably delimiting *Mazama* species. As discussed previously, karyotypic evidence has consistently proven decisive in defining species boundaries within the genus (Abril and Duarte 2008; Cursino et al. 2014; Peres et al. 2021; Sandoval et al. 2024). Future research on these recently recognized lineages should include detailed chromosomal analyses. Such integrative approaches are crucial to resolve the taxonomic status of other brocket deer that were described after *M. tschudii*. Therefore, morphological, cytogenetic, and phylogenetic data presented here provide evidence that *M. tschudii* (Wagner, 1855) should be recognized as a valid species within the genus *Mazama*.

### ﻿*Mazama tschudii* (Wagner, 1855)

Despite the existence of two historical names for Peruvian brocket deer – *Mazama tschudii* and *Mazama whitelyi* – Wagner’s original description of *tschudii* in 1855 refers to a small, gray or brown brocket from the western slopes of the Peruvian Andes. This matches both the phenotype and the biogeographic context of our collected specimen, which also originates from the inter-Andean valleys on the arid western versant. In contrast, *M. whitelyi* was described in 1873 by Gray based on the skull of a juvenile specimen from the Kosñipata Valley (Fig. 1), located on the humid eastern flank of the Andes, a region characterized by montane cloud forests and transitional Amazonian habitats. These biomes are ecologically and faunistically distinct from the region associated with *M. tschudii*. Additionally, *M. whitelyi* has historically been treated as a synonym of *Mazama americana*, a medium-sized, reddish brocket deer – unlike *M. tschudii*, which has consistently been associated with smaller body size and grayish coloration. Given these distinctions, and the fact that *M. tschudii* has nomenclatural priority over *whitelyi*, it is necessary to validate Wagner’s name. Our specimen closely matches the original description and locality of *M. tschudii*, and the name does not correspond to any currently recognized valid species. Therefore, its validation fulfills the criteria of the ICZN and contributes to stabilizing the taxonomy of South American brocket deer.

**Etymology.** Named by Johann Andreas Wagner in 1855 in honor of the Swiss naturalist and explorer Johann Jakob von Tschudi. Tschudi was known for his extensive travels and scientific studies in South America, particularly in Peru, where he conducted research on the region’s fauna, flora, geography, and indigenous cultures. His work significantly contributed to the understanding of Andean biodiversity.

**Diagnosis.***Mazama tschudii* is a small to medium-sized deer with a brownish coat and a distinct dark dorsal stripe. It has a dark gray neck contrasting with paler flanks and a pale ventral region with white tail hairs. Limbs show brown, reddish, and gray tones, and the ears are dark with a white, hairless inner base. Facial markings include a white nasal patch, a frontal tuft, and yellowish details around the eyes.

Compared to other Neotropical deer species, *Mazama tschudii* exhibits a unique set of morphological traits. Unlike *Passalites nemorivagus*, which has a more uniform coloration, *M. tschudii* is characterized by a strong contrast between the dark gray neck and the rest of the body, as well as a well-defined dorsal stripe. In contrast to *Subulo gouazoubira*, which has a more reddish-gray coat, *M. tschudii* possesses darker and more defined facial and dorsal markings. *Mazama tschudii* differs from *Mazama temama* by its paler ventral areas and distinctive white tail hairs, whereas *M. temama* generally exhibits a darker red overall coloration. Unlike *Mazama rufina*, a highland species with denser and redder fur and a more pronounced dark mask, *M. tschudii* has a brownish pelage with a distinct dorsal stripe. Compared to the larger *M. americana*, which has a more uniform reddish tone, *M. tschudii* is smaller and presents more contrasting markings and distinctive facial features. These characteristics, including the presence of a white nasal patch and frontal tuft, further differentiate *M. tschudii* from its Neotropical relatives, highlighting its unique morphological identity within the group.

#### ﻿Synonymy

*Cervus tschudii* Wagner, 1855, Schreber’s Saugthiere 386–387. Original description.

Cervus (Subulo) tschudii Wagner, 1855, loc. cit.

Cervus (Subulo) simplicicornis major Wagner, 1855, loc. cit.

*Doryceros tschudii* – Fitzinger 1873, Akad. Wiss. Wien, 68, part I, 360. New combination.

*Mazama tschudii* (Wagner, 1855) – Lydekker 1898, Deer of all Lands: 305. First use of the current combination.

*Mazama tschudii* – Allen 1915 Bull. Amer. Mus. Nat. Hist., XXXI, p. 74.

*Mazama gouazoubira tschudii* – Cabrera 1960. Catálogo de los mamíferos de América del Sur. Ver. Mus. Arg. Bern. Riv., 4: 341.

**Neotype designation.** Johann Andreas Wagner (1855) described *Mazama tschudii* based on a specimen from the western Andes in the coastal region of Peru. However, specific details about the exact collection locality and the existence of a type specimen were not provided, leaving the precise type locality ambiguous. Extensive efforts were made to locate the original holotype or any original topotypic material of *Cervus tschudii* in European zoological collections potentially associated with Wagner’s work or with the collections of Johann Jakob von Tschudi. Nevertheless, no records were found indicating that such material was deposited and there is no institution mentioned in historical sources nor referenced in museum databases as holders of Wagner’s original specimens. In the absence of a designated holotype or detailed locality information, establishing a type locality for *Mazama tschudii* requires careful consideration of historical records and current taxonomic practices. One approach is to designate a neotype, a specimen selected to serve as the type specimen when the original is lost or not designated. This process is governed by Article 75 of the International Code of Zoological Nomenclature (ICZN), which outlines the conditions under which a neotype can be designated, including the necessity to clarify taxonomic status and the unavailability of original type material. Aiming to designate a neotype and establish a type locality for *Mazama tschudii*, a specimen was collected from a location that aligns with Wagner’s original description in the western Andes in the coastal region of Peru. Specifically, a male specimen was collected on 22 January 2020, from La Ramada, Salas District, Lambayeque Province, Peru, at an altitude of 1,510 meters above sea level (6°24'57"S, 79°43'1"W). This locality corresponds with the general area described by Wagner and provides a precise geographic reference. Designating this specimen as the neotype would not only establish La Ramada as the type locality but also provide a concrete reference for future taxonomic and conservation studies of *Mazama tschudii*. Such a designation would resolve existing ambiguities regarding the identity and origin of the species, facilitating more accurate phylogenetic analyses. By clarifying the identity of the species and type locality, this action provides the taxonomic foundation necessary for subsequent research on its distribution, conservation status, and long-term management.

## ﻿Conclusion

The collection and assignment of a neotype for *Mazama tschudii* (Wagner, 1855) enabled a comprehensive reevaluation of this historically overlooked taxon. Morphological characterization revealed the specimen with a uniform dark brown pelage and diagnostic body measures that distinguish *M. tschudii* from other Neotropical deer. Cytogenetic analysis revealed a unique chromosomal composition, markedly distinct from other *Mazama* species, including *M. temama* sensu stricto, its sister taxon within a well-supported monophyletic clade based on complete mitochondrial genomic data. This marked karyotypic divergence underscores the presence of a genetically and likely reproductively isolated lineage. Thus, the integrative approach combining morphological, cytogenetic, and molecular evidence provides robust taxonomic resolution and confirms that the brocket deer from the western Cordillera of Peru represents a valid and distinct species within the genus *Mazama*.

## ﻿Additional information

### Use of AI

No use of AI was reported.

### Author ORCIDs

Eluzai Dinai Pinto Sandoval https://orcid.org/0000-0001-6849-7373

Eduard José Hernández Guevara https://orcid.org/0000-0002-5919-8408

Agda Maria Bernegossi https://orcid.org/0000-0003-0369-1858

Pedro Henrique Faria Peres https://orcid.org/0000-0002-3158-0963

Renato Caparroz https://orcid.org/0000-0003-4883-4023

José Maurício Barbanti Duarte https://orcid.org/0000-0002-7805-0265

### Data availability

All of the data that support the findings of this study are available in the main text or Supplementary Information.

## Supplementary materials

Supplementary material 1 Body measurements in centimeters (cm) of a male topotype of *Mazama tschudii* (Wagner, 1855), collected in collected in Lambayeque, Northwest Andean Cordillera of Peru Data type docx https://binary.pensoft.net/file/1498852This dataset is made available under the Open Database License (http://opendatacommons.org/licenses/odbl/1.0/). The Open Database License (ODbL) is a license agreement intended to allow users to freely share, modify, and use this Dataset while maintaining this same freedom for others, provided that the original source and author(s) are credited.Eluzai Dinai Pinto Sandoval, Eduard José Hernández Guevara, Agda Maria Bernegossi, Pedro Henrique Faria Peres, Renato Caparroz, José Maurício Barbanti Duarte

Supplementary material 2 Skull measurement of a male topotype of *Mazama tschudii* (Wagner, 1855), collected in collected in Lambayeque, Northwest Andean Cordillera of Peru Data type docx https://binary.pensoft.net/file/1498853This dataset is made available under the Open Database License (http://opendatacommons.org/licenses/odbl/1.0/). The Open Database License (ODbL) is a license agreement intended to allow users to freely share, modify, and use this Dataset while maintaining this same freedom for others, provided that the original source and author(s) are credited.Eluzai Dinai Pinto Sandoval, Eduard José Hernández Guevara, Agda Maria Bernegossi, Pedro Henrique Faria Peres, Renato Caparroz, José Maurício Barbanti Duarte
