# Supplementary material for: ﻿Mazama tschudii (Wagner, 1855), forgotten by science, re-emerges as a new genetic lineage of Neotropical deer with a proposed neotype (Artiodactyla, Cervidae)
Source: Zookeys. 2025 Dec 23;1265:25–47. doi: 10.3897/zookeys.1265.157429 (PMC12754598; doi:10.3897/zookeys.1265.157429)
Supplement: Supplementary material 1 — Body measurements in centimeters (cm) of a male topotype of Mazama tschudii (Wagner, 1855), collected in collected in Lambayeque, Northwest Andean Cordillera of Peru [file zookeys-1265-025_article-157429__-s001.docx]

**Supplementary Table 1.** Body measurements in centimeters (cm) of a male topotype of *Mazama tschudii* (Wagner, 1855), collected in collected in Lambayeque, Northwest Andean Cordillera of Peru.

| **Character (cm)** | **T431** | **Caráter (cm)** | **T431** |
| --- | --- | --- | --- |
| Head length | 22,5 | Body length | 74,7 |
| Head width | 7,5 | Abdomen diameter | 57,8 |
| Ear length | 9,6 | Right testicle length | 5,3 |
| Between eyes | 4,4 | Left testicle length | 5,2 |
| Mandible | 6,3 | Right testicle diameter | 2,1 |
| Neck diameter | 24,2 | Left testicle diameter | 2,2 |
| Thorax diameter | 50,2 | Metatarsus length | 19,1 |
| Height | 51 | Tail length | 7,5 |
| Metacarpus length | 11 | Mass (Kg) | 13,6 |
