# Supplementary material for: ﻿Mazama tschudii (Wagner, 1855), forgotten by science, re-emerges as a new genetic lineage of Neotropical deer with a proposed neotype (Artiodactyla, Cervidae)
Source: Zookeys. 2025 Dec 23;1265:25–47. doi: 10.3897/zookeys.1265.157429 (PMC12754598; doi:10.3897/zookeys.1265.157429)
Supplement: Supplementary material 2 — Skull measurement of a male topotype of Mazama tschudii (Wagner, 1855), collected in collected in Lambayeque, Northwest Andean Cordillera of Peru [file zookeys-1265-025_article-157429__-s002.docx]

**Supplementary Table 2.** Skull measurement of a male topotype of *Mazama tschudii* (Wagner, 1855), collected in collected in Lambayeque, Northwest Andean Cordillera of Peru. In millimeters (mm) following von den Driesch (1976)

| **Character (mm)** | **T431** | **Character (mm)** | **T431** | **Character (mm)** | **T431** |
| --- | --- | --- | --- | --- | --- |
| **TL** | 174,7 | **AK** | 120,6 | **GBBP** | 58,9 |
| **CBL** | 168,28 | **GLN** | 45,8 | **GBFM** | 16,15 |
| **BL** | 154,9 | **SLFL** | 86,1 | **GNB** | 56,03 |
| **SSL** | 104,2 | **OPL** | 79,04 | **GBAO** | 73,15 |
| **PR** | 50,7 | **LLP** | 40,2 | **LBBO** | 39,5 |
| **BCA** | 35,02 | **LCR** | 55,87 | **ZB** | 72,6 |
| **BFA** | 74,5 | **LMR** | 29,73 | **GBAN** | 15,1 |
| **VL** | 74,7 | **LPR** | 26,7 | **GBAP** | 22,7 |
| **MFL** | 109,8 | **GILO** | 30,41 | **GPB** | 50,91 |
| **LN** | 96,1 | **GIHO** | 29,06 | **BHPSN** | 43,22 |
| **LR** | 138,05 | **GMB** | 71,19 |  |  |
| **LP** | 164,4 | **GBOC** | 35,41 |  |  |

TL= total length, CBL= condilobasal length, BL= basal length, SSL= short skull length, PR= premolare – prostion, BCA= basecranial axis, BFA= basefacial axis, VL= viscerocranium length, MFL= median frontal length, LN= lambda – nasal, LR= lambda – Rhinion, LP= lambda – prostion , AK= akrokranium, GLN= greatest length of the nasals, SLFL= short lateral facial length, OPL= oral palatal length, LLP= lateral length of the premaxilla, LCR= length of the cheektooth row, LMR= length of the molar row, LPR= length of the premolar row, GILO= greatest inner length of the orbit, GIHO= greatest inner height of the orbit, GMB= greatest mastoid breadth, GBOC= greatest breadth of the occipital condyles, GBBP= greatest breadth at the bases of the paraoccipital, GBFM= greatest breadth of the foramen magnum, HFM= Height of the foramen magnum: Basion - Opisthion, GNB= Greatest neurocranium breadth, LFB= Least frontal breadth, GBAO= greatest breadth across the orbits, LBBO= least breadth between the orbits, ZB= zygomatic breadth, GBAN= greatest breadth across the nasals, GBAP= greatest breadth across the premaxillae, GPB= greatest palatal breadth, BHPSN= Basion – The highest point of the superior nuchal crest.
